# Immune-related adverse events with renal colic as the main manifestation: a case report of sintilimab-induced ureteritis/cystitis treated by ureteral stent and review of the literature

**DOI:** 10.3389/fimmu.2024.1501415

**Published:** 2024-12-23

**Authors:** Jiaxiang Ji, Chin-Hui Lai, Xiaowei Zhang, Hao Hu

**Affiliations:** ^1^ Department of Urology, Peking University People’s Hospital, Beijing, China; ^2^ The institute of applied lithotripsy technology, Peking University, Beijing, China

**Keywords:** immune checkpoint inhibitors (ICIs), immune-related adverse events (irAEs), sintilimab, ureteritis/cystitis, ureteral stent

## Abstract

Immune checkpoint inhibitors (ICIs) have revolutionized the treatment of systemic cancer therapy. During disinhibiting the antitumor responses of immune system, ICIs may also cause unique immune-related adverse events (irAEs) which could affect any organ. Here, we report a rare case of sintilimab-induced ureteritis/cystitis in a 55-year-old male undergoing neoadjuvant chemo-immunotherapy for gastric cancer. The patient presented with severe renal colic, hematuria, and hydronephrosis. Antibiotic antispasmodic and nonsteroidal anti-inflammatory drug treatment failed to alleviate symptoms. The application of corticosteroid was negated for recent extirpative surgery under plan. Bilateral ureteral stenting effectively resolved the pain and improved renal function. The patient later successfully underwent laparoscopic radical gastrectomy with significant tumor regression in postoperative pathology. This case highlights ureteral stenting as a potential corticosteroid-free treatment option for ICI-induced ureteritis/cystitis.

## 1 Introduction

As Immune checkpoint inhibitors (ICIs) become more prevalent in cancer therapy, immune-related adverse events (irAEs) gradually attract the attention of oncologists (1). Essentially, irAEs stem from the overactivity of immune system, which induces off-target autoimmune response besides on-target antitumor effect. IrAEs can theoretically affect any organ and in the worst scenario, become life-threatening (2).

As a member of ICIs, sintilimab (Tyvyt^®^) is a fully humanized immunoglobulin G4 monoclonal antibody that binding to PD-1, which in turn selectively blocks the interaction between PD-1 and its two known ligands, PD-L1 and PD-L2 (3). As a result, it could facilitate T-cell activation and proliferation, thus promoting immunoactivity against malignancies. Sintilimab has shown efficacy in the treatment of multiple malignancies including lymphoma, lung cancer, hepatocellular carcinoma, esophageal cancer and gastric cancer (4, 5).

Despite favorable outcomes, it could also lead to a wide spectrum of irAEs (6, 7). However, sintilimab-associated irAEs affecting the urinary tract have rarely been reported and discussed (8). The treatment of immune-related cystitis/ureteritis, like other irAEs, depends heavily on steroids. While most irAEs are steroid-sensitive, there are notable drawbacks. Corticosteroid could indiscriminately suppress immune function, and potentially, inhibit antitumor immune response. And large dose corticosteroid administration inevitably carries other side effects, especially in certain circumstances such as facing impending major surgery (9). The use of steroid pulse therapy has been associated with a number of potential adverse effects, including increased risk of infection, impaired wound healing, and alterations in glucose metabolism (10, 11). Here, we report a case of sintilimab-induced ureteritis/cystitis during neoadjuvant chemo-immune therapy which was successfully treated by ureteral stenting in a corticosteroid-free way.

## 2 Case presentation

A 55-year-old male patient with history of urolithiasis was admitted to Peking University People’s Hospital due to abdominal discomfort. Biopsy under gastric scope indicated poorly differentiated gastric adenocarcinoma. Thoracoabdominal pelvic enhanced computed tomography revealed gastric mass and the tumor was staged as cT4aN2M0. The immunohistochemical examination of the gastric mass indicated CK(+), MLH1(+), MSH2(-), MSH6(+), PMS2(+), C-erbB-2(0), Ki67 (20%+). The expression of PD-L1 is negative in tumor cells. His performance score (PS) was 1 (PS ranged from 0 to 6, and the lower value indicated better physical condition).

The patient began to receive neoadjuvant chemo-immunotherapy on April 1, 2024. He was administered with Oxaliplatin (IV, 200 mg at Day 1), Tegafur (po, 40mg/bid from Day 1-Day 14) and sintilimab (IV, 200mg at Day 1) for 3 courses.

**Figure 1:**
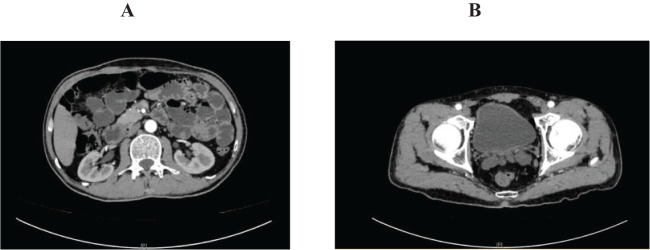
Abdominal pelvic enhanced computed tomography on March 13. **(A)** renal pelvis; **(B)** bladder. **(A)** showed renal pelvis and upper ureter before sintilimab treatment; **(B)** showed bladder before sintilimab treatment.

**Figure 2:**
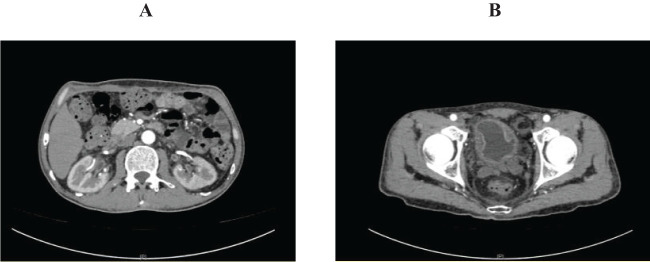
Abdominal pelvic enhanced computed tomography on June 7. **(A)** showed mild hydronephrosis, dilation renal pelvis and thickened ureter wall; **(B)** showed thickened bladder wall and poor bladder filling.

On June 5, 15 days after the last treatment course, the patient complained of progressively exacerbated left renal colic and ongoing gross hematuria for one week. He had percussion pain (+) in left renal and ureter area. Urinalysis showed red blood cells (5500/µl) and white blood cells (670/µl). His white blood cells in routine blood examination were 2.74 (normal 3.5-9.5) ×10^9^/L. Repeated urine culture was all negative. Also, serum creatinine (Scr) level elevated to 126 µmol/L (baseline Scr 70 µmol/L). Urinary ultrasonography showed mild hydronephrosis and dilation of ureter on the left and a thickened bladder wall. Recurrent urolithiasis was suspected. Thoracoabdominal enhanced computed tomography in June 9, 2024 showed mild reduction of the tumor. However, thickened hepative capsule was identified and seeding metastasis was suspected. Thus, the treatment response was evaluated as progressive disease (PD). Also, CT revealed, compared to images before neoadjuvant chemo-immunotherapy (**Figure 1**), poor bladder filling, slightly thickened bladder and ureter wall, slightly enlarged left kidney and no obvious ureteral calculus (**Figure 2**). A multidisciplinary team (MDT) meeting was organized, and the diagnosis of sintilimab-induced cystitis/ureteritis was made after exclusion the evidence of urinary infection, urolithiasis and tumor metastasis.

The patient received phloroglucinol, levofloxacin and flurbiprofen axetil. However, all of them failed to relieve left renal colic and even contralateral renal colic occurred.

As Laparoscopic radical gastrectomy was proposed, the application of systemic corticosteroid therapy was negated. For fear of deteriorated hydronephrosis and renal function, bilateral ureteral stent was preemptively inserted. Cystoscopy revealed swollen and ulcerative bladder mucosa. Beyond physician’s expectation, renal colic disappeared immediately. In the following days, hematuria was also slightly alleviated. His Scr dropped to 66 mmol/l on 14 August 2024.

**Figure 3:**
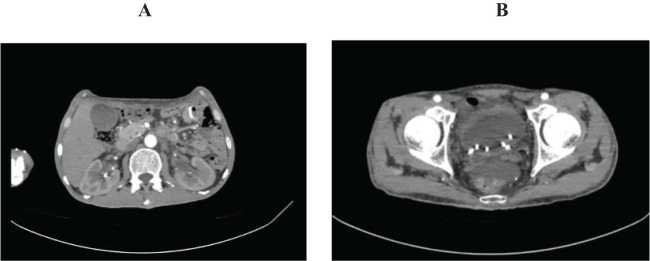
Abdominal pelvic enhanced computed tomography on August 14. **(A)** showed reduced dilatation of renal pelvis and ureter. **(B)** showed reduced swelling bladder mucosa.

**Figure 4:**
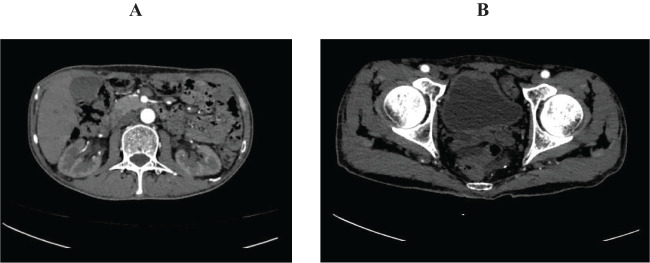
Abdominal pelvic enhanced computed tomography on November 19. **(A)** showed normal renal pelvis and ureter. **(B)** showed well filled bladder with normal mucosa.

**Figure 5:**
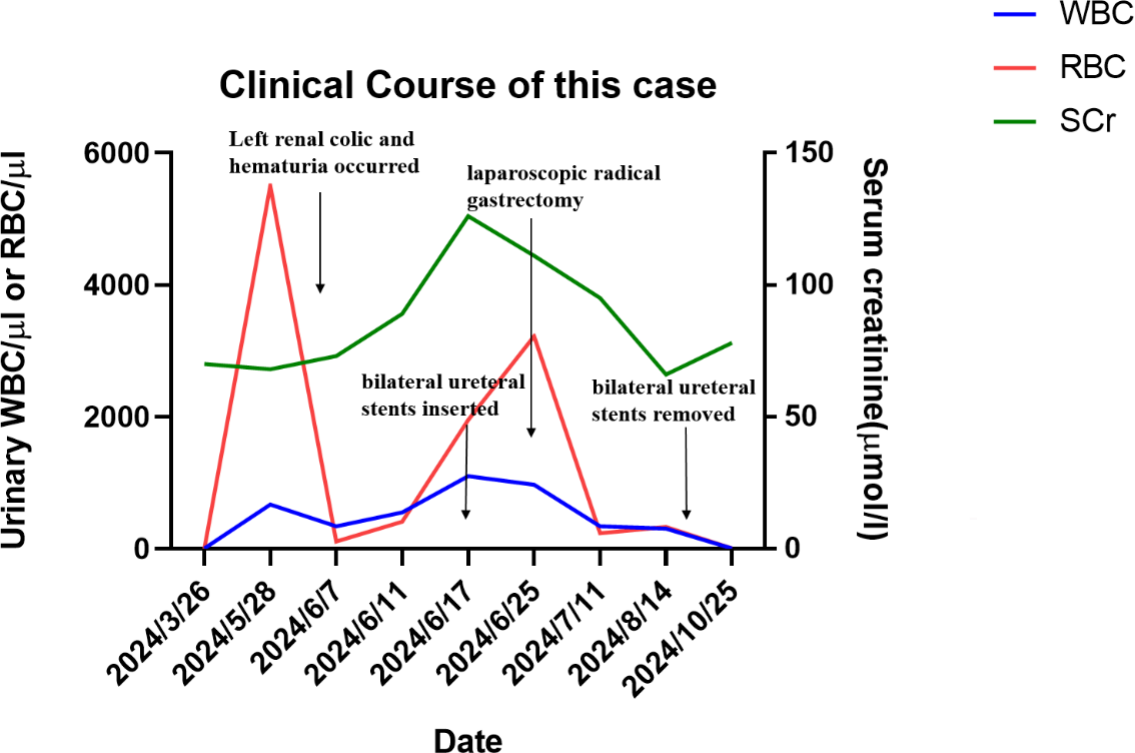
The timeline of the clinical course of this case.

The patient underwent laparoscopic radical gastrectomy after a week. Postoperative pathology indicated poorly differentiated adenocarcinoma of submucosa muscular invasion. Tumor regression grade was evaluated as TRG1. Pathology stage was classified as ypT2N1M0. Thoracoabdominal pelvic enhanced computed tomography on August 14 revealed no obvious ureteritis/cystitis and gross hematuria was also significantly alleviated (**Figure 3**). Bilateral ureteral stents were removed three months later. CT scan in November showed normal bladder mucosa (**Figure 4**). The patient is currently undergoing adjuvant chemotherapy and there is no further complaint of renal colic. The timeline of the treatment course was summarized in **Figure 5**.

## 3 Discussion

ICIs represent a class of antineoplastic agents that specifically target immune checkpoint with inherent immunosuppressive functions. These agents function by enhancing the antitumor activity of lymphocytes, thereby providing substantial therapeutic benefits for patients with advanced malignancies. Nevertheless, despite their demonstrated antitumor efficacy, ICIs can compromise immune tolerance, resulting in damage to normal tissues across various organ systems. Such adverse effect is referred to as irAEs.

Studies regarding comparison of irAEs among different ICIs are rare. It is now found that the toxicity of ipilimumab, a CTLA4 blocker, is dose-dependent, while that of PD1/PD-L1 inhibitors does not seem to be dose-dependent (12, 13). Also, the incidence of high grade irAEs seems to be lower in patients treated by PD1/PD-L1 inhibitors, occurring in 15–20% of patients, which is lower than the toxicity observed with single-agent anti-CTLA4 therapy, with severe irAEs incidence of 25-50% in patients with melanoma treated by ipilimumab (12, 14). Given the expanding clinical application of ICIs and the concomitant rise in the incidence of severe adverse events, it is imperative to attain a comprehensive understanding of these irAEs.

Up to now, sintilimab has shown efficacy in the treatment of multiple malignancies including lymphoma, lung cancer, hepatocellular carcinoma, esophageal cancer and gastric cancer. However, irAEs also occur during sintilimab treatment (6, 7). Although it could affect any organ, irAEs of the urinary system is rarely induced by sintilimab. A total of 15 cases of ICIs-induced ureteritis/cystitis have been reported so far, with most patients (14/15) treated by PD-1 antibody (8, 15–25) (**Table 1**). Among them, nearly half (7/15) were treated by nivolumab, the others including sintilimab(3/15), pembrolizumab(2/15), tislelizumab(2/15) and atezolizumab(1/15).

**Table 1:** Clinical information of the case reports of ICIs-induced ureteritis/cystitis.

ICIs	Age/Gender	Carcinoma/stage	Symptoms	Treatment	Outcome
Nivolumab	61/male (25)	Melanoma/IV	Intense bladder pain, urgency, and nocturia after 2 cycles	Prednisolone,0.5mg/kg/d	The symptoms subsided after 7 days
	51/male (21)	Small cell lung cancer/IV	Pollakisuria after 5 cycles	Methylprednisolone, 80mg/iv/bid	Symptoms resolved after 3 days
	50/male (18)	Lung squamous cell carcinoma/IV	Pollakisuria, odynuria after 7 cycles	Prednisolone, 1mg/kg/day	Immediately alleviated
	60/male (18)	Lung squamous cell carcinoma/IV	Pollakisuria, odynuria after 12 cycles	Discontinuation of ICIs	Symptoms subsided immediately
	47/male (20)	Pulmonary adenocarcinoma/IV	Pollakiuria and micturition pain after 18cycles	No	Subsided after bladder biopsy
	62/male (17)	Lung squamous cell carcinoma/IV	Odynuria, pollakiuria, and macroscopic hematuria after 3 cycles	Methylprednisolone, 500 mg/day	Resolved quickly
	48/male (36)	Intrahepatic cholangiocarcinoma	Bladder irritation after 3 cycles	Steroid hormones,2 mg/kg/day	Resolved quickly
Sintilimab	53/male (8)	Pulmonary adenocarcinoma/IVB	Hematuria, pollakiuria and painful micturition after 3 cycles	Methylprednisolone, (80mg,1mg/kg/d)	Symptoms obviously alleviated after two days
	56/male (22)	Gastric carcinoma	Pollakiuria, micturition pain, and urinary incontinence after 5 cycles	Chai-Ling-Tang	Symptoms subsided immediately
	62/female (16)	Gastric carcinoma/IV	Urinary irritation after 3 cycles	Methylprednisolone, 60 mg/day	Symptoms relieved after 1 week
Pembrolizumab	78/female (19)	Lung adenocarcinoma/IV	Pollakiuria, nocturia, odynuria	Prednisolone, 25 mg/day	Symptoms improved after 19 days
	56/male (23)	Lung squamous carcinoma/III	Pollakiuria, urgency and odynuria after 6 cycles	Methylprednisolone, 40 mg/3 days	Symptoms relieved after 3 days
Tislelizumab	49/male (16)	Esophageal carcinoma/IV	Gross hematuria, pollakiuria, odynuria after 6 cycles	Methylprednisolone 60 mg/day	Symptoms relieved after 3 days
	27/male (15)	Thymic cancer/IV	Bellyache after 6 cycles	Methylprednisolone 1 mg/kg	Symptoms relieved after 3 days
Atezolizumab	67/female (37)	Breast cancer/IV	Frequency, odynuria after 4 cycles	Prednisolone 40 mg/day	Symptoms disappeared after two days

The main manifestations of prior patients were lower urinary tract symptoms (LUTS), including frequent urination, dysuria, odynuria, nocturia, hematuria and incontinence. Only two of them had low back pain, however, the pain was relatively mild. As a comparison, the main manifestation of our patient was refractory renal colic, which was different from other immune-induced ureteritis/cystitis cases. In contrast, LUTS was relatively mild.

Laboratory analyses frequently demonstrate a marked elevation in red blood cells (RBCs) and white blood cells (WBCs) within the urine, accompanied by elevated serum creatinine (Scr) levels. Negative findings in urine culture and cytology can aid in excluding urinary tract infections. Prior to confirming the diagnosis of immune therapy-related ureteritis/cystitis, it is imperative to rule out other potential etiologies presenting with analogous symptoms, such as urolithiasis or tumor infiltration. Cystoscopic examination could reveal diffuse erythema of the bladder and ureteral mucosa. CT scans and magnetic resonance imaging (MRI) are valuable diagnostic tools for evaluating structural alterations in the ureters, including congestion, edema, strictures, and obstructions. Common findings on CT and MRI typically include bilateral dilation of the upper urinary tract and thickening of the ureter/bladder wall.

The definitive diagnosis of ICIs-induced ureteritis/cystitis is contingent upon pathological examination. Bladder and ureter mucosal biopsies generally demonstrate substantial lymphocytic infiltration, marked by elevated expression of CD8+ and CD3+ T lymphocytes, as well as T-cell intracellular antigen-1 (TIA-1). This infiltration is frequently associated with high levels of programmed death-ligand 1 (PD-L1) expression. Importantly, the urothelium is devoid of tumor cells. In this case, the patient was clinically diagnosed with ICIs-induced ureteritis/cystitis for several reasons. First, he underwent three courses of ICIs treatment. Second, CT and laboratory test indicated the typical presentation of ureteritis/cystitis. Third, urolithiasis, infection or urinary malignancy were all precluded.

At present, the management of irAEs predominantly relies on clinical expertise and is conducted in accordance with the grading criteria established by the Common Terminology Criteria for Adverse Events (CTCAE). For grade 1 adverse events, specific therapeutic interventions are generally unnecessary, allowing for the continuation of immunotherapy. In the case of grade 2 adverse events, it is recommended to suspend ICIs treatment until symptomatic improvement is observed, with some patients potentially necessitating corticosteroid therapy. Patients who experience grade 3 or 4 adverse events typically require corticosteroid treatment and may need to either temporarily or permanently discontinue ICIs therapy.

However, there are now no established common criteria for grading immune-related ureteritis/cystitis. Generally, when the patient needs hospitalization or invasive procedure, it should be classified as grade 3 or higher. Most patients with ICIs-induced ureteritis/cystitis were steroid-sensitive. But there is long-lasting concern over the immunosuppressive effect of steroid which some believe could jeopardize the therapeutic effect of immunotherapy (26, 27). Due to concerns of inhibiting immune-mediated tumor regression, the ongoing use of steroid is a common exclusion criterion of ICIs-related clinical trial (28, 29). Thus, oncologists often face a dilemma in the systemic application of large dose corticosteroid, which might impede ICIs efficacy. As to this case, the patients didn’t find tumor regression in CT, which prompt surgeons to perform extirpative surgery as soon as possible. In such scenario, the administration of large dose steroid could also have a negative impact on the postoperative recovery or even facilitate tumor dissemination (30, 31).

In this case, the patient presented with severe renal colic as the main symptom. NSAIDs and spasmolytic agents all failed to alleviate severe pain effectively. Indwelled ureteral stents, however, eliminated the pain immediately. A possible explanation is that the indwelled ureteral stent could lower intrarenal pressure, decrease or even stop ureter peristalsis (32). This is both unique and meaningful. Unlike irAEs of other organs, ICIs-induced ureteritis/cystitis is usually localized without life-threatening risk. Compared to systemic steroid application, the placement of ureteral stent, as a local therapy, could alleviate severe renal colic, protect renal function and at the same time, mitigate the risk of systemic steroid. Another interesting point is that irAEs has been long proposed as a predictor for superior ICIs response (33, 34). Previous immune-related ureteritis/cystitis most occurred in patients of metastatic disease, thus failed to capture the exact pathological response. In this case, the patient underwent laparoscopic radical gastrectomy after the occurrence of immune-related ureteritis/cystitis. Although tumor remission is not observed in thoracoabdominal enhanced CT, pathological examination did find superior therapeutic response (ypT2N1M0) and tumor regression (TRG1).

In addition, ureteritis/cystitis was significantly relieved in a steroid-free fashion. This is enlightening, as previous cases of ICIs-induced ureteritis/cystitis were mostly treated with large dose corticosteroid. And large dose corticosteroid inevitably brings about many undesired side effects, which is why corticosteroid is not administered during this treatment process. It is reported that patients not given dexamethasone had better three-year survival outcomes compared with patients given dexamethasone perioperatively (9). And for patients underwent a major procedure like radical gastrectomy, the application of large dose corticosteroid often brought more harm than good, resulting in delayed wound healing, hyperglycemia and infection. According to this case, ureteritis/cystitis induced by ICIs is self-limited and could gradually vanish without medical therapy.

Here, we report a case of sintilimab-induced ureteritis/cystitis. The case is important for several reasons, especially considering the rarity of such irAEs. First, for patients of ICIs-induced ureteritis/cystitis, ureteral stenting may be a feasible substitute for large dose corticosteroid administration. Unlike liver, lung or heart, which are vital in maintaining homeostasis, the biological function of ureter/bladder is rather simple, i.e., drainage and storage of urine. Thus, the main therapeutic management of ICIs-induced ureteritis/cystitis should be directed to protect renal function and relieve irAEs-related symptoms. In this respect, ureteral stenting could play an important role, which, as a local therapy, could effectively drain the renal pelvis, protect renal function and even relieve renal colic. This is unique in the treatment of irAEs, as no feasible local therapy exists for irAEs of any other organ. Second, the swelling of ureteral and bladder wall could subside without corticosteroid. In previous reports of ureteritis/cystitis, most cases were treated with corticosteroid. As corticosteroid is indeed the major drug dealing with irAEs, it could also bring about many undesired side effects. In this case, when facing an impending radical surgery, there is a trade-off between the benefits of the drug and the risk of side effects. Considering the low life-threatening risk of ureteritis/cystitis and uncertain risk of perioperative administration of large dose corticosteroid, the surgeon and patients adopted a wait-and-watch strategy and chose ureteral stenting as a substitute. Third, we must emphasize that the role of corticosteroid in irAEs is still irreplaceable in the foreseeable future. If ureteritis/cystitis does not fade away over time, the administration of large dose corticosteroid must be mooted. We must also acknowledge the risk of delayed corticosteroid, which might result in longer hospitalization, steroid taper failure, longer duration of symptoms, as previous study supported the use of immunosuppressive therapy with steroids earlier rather than later (35).

## 4 Conclusions

This article presents a case of ureteritis/cystitis induced by immunotherapy, characterized predominantly by severe pain and treated successfully by ureteral stenting. The article conducts a comprehensive review of pertinent case reports and literature, synthesizing the potential pathogenesis, diagnostic criteria, and therapeutic approaches for immune-related ureteritis. This case suggests that in patient of ICIs-induced ureteritis/cystitis, ureteral stenting could protect renal function and relieve renal colic effectively. And ureteritis/cystitis could subside without systemic corticosteroid. Of note, this conclusion needs the support of more clinical data.

## Data Availability

The raw data supporting the conclusions of this article will be made available by the authors, without undue reservation.
